# Quercetin Differentially Modulates MTT-Derived Cellular Metabolic Activity and Apoptosis-Related Gene Expression in A549 and BEAS-2B Cells

**DOI:** 10.3390/metabo16100740

**Published:** 2026-10-01

**Authors:** Deniz Özkan Vardar, Tuğçe Duran, Merve Aydın

**Affiliations:** 1 Pharmacy Services Program, Vocational School of Health Services, Lokman Hekim University, 06510 Ankara, Türkiye; 2 Department of Medical Genetics, Faculty of Medicine, KTO Karatay University, 42020 Konya, Türkiye; 3 Department of Medical Microbiology, Faculty of Medicine, Erzincan Binali Yıldırım University, 24100 Erzincan, Türkiye

**Keywords:** quercetin, secondary plant metabolite, cancer, A549, BEAS-2B, cellular metabolic activity, apoptosis-related gene expression, natural compound

## Abstract

Background/Objectives: Quercetin is a dietary flavonoid with potential anticancer activity, but comparative effects in malignant and non-malignant lung-derived epithelial cells remain incompletely characterized. Methods: A549 and BEAS-2B cells were exposed to quercetin (1–250 µM) for 24, 48, and 72 h, and MTT-derived cellular metabolic activity was measured. Mean normalized concentration–response curves from three independent biological experiments (*n* = 3) were fitted with four-parameter logistic models, and Greenhouse–Geisser-corrected cell line × concentration × time interactions were tested. Apoptosis-related gene expression was assessed by qRT-PCR after exposure to 45 µM quercetin for 48 h; 45 µM was not a discrete concentration in the MTT series and was close to the directly tested 40 µM condition. Results: Apparent MTT-derived IC_50_ values were 58.28 µM (95% CI 44.18–74.81) for A549 and 121.22 µM (89.50–164.66) for BEAS-2B at 48 h, and 2.73 µM (2.31–3.67) and 1.49 µM (1.27–1.77), respectively, at 72 h. Cell line × concentration interactions were significant at all exposure times, as was the cell line × concentration × time interaction; transcriptional profiles differed descriptively between cell lines. Conclusions: The fitted IC_50_ ordering reversed between 48 and 72 h, so a stable cancer-selective response is not supported. MTT reflects metabolic activity rather than cell death, and transcript changes do not establish functional apoptosis.

## 1. Introduction

The discovery and characterization of bioactive natural compounds remain important components of anticancer drug development. Plant-derived secondary metabolites are of particular interest because they can influence cellular redox balance, proliferation, metabolism-associated processes, and cell-death signaling through multiple molecular targets. Polyphenols are abundant in plant-based foods and beverages, and epidemiological and experimental studies have associated polyphenol-rich dietary patterns with reduced cancer risk [1,2,3,4,5,6,7,8]. Quercetin is a widely distributed flavonol and secondary plant metabolite found in fruits, vegetables, teas, and medicinal plants [9]. Its biological effects include modulation of mitochondrial integrity, caspase signaling, redox homeostasis, and responses to chemotherapeutic agents [10,11,12,13]. These properties have supported continued investigation of quercetin as a natural compound of potential relevance to antineoplastic drug development.

Cellular metabolic status and programmed cell-death signaling are closely interconnected in cancer biology. Apoptosis contributes to tissue homeostasis and elimination of damaged cells and is regulated through coordinated mitochondrial pathways involving caspases, *TP53*, and BCL-2 family proteins. Flavonoids have been reported to modify these pathways and influence the balance between pro-survival and pro-death signaling [14,15,16,17]. Accordingly, evaluating a metabolic-response endpoint together with apoptosis-related transcriptional markers can provide complementary information on how a bioactive compound affects cellular function, while recognizing that neither endpoint alone establishes a complete mechanism of action.

A549 lung adenocarcinoma cells are widely used as an in vitro model of non-small cell lung cancer (NSCLC), whereas BEAS-2B cells provide an immortalized non-malignant bronchial epithelial comparator. Previous studies have shown that quercetin can modulate apoptosis-related signaling in lung cancer models, including A549 cells, and comparative work has applied common nominal quercetin concentrations across malignant and non-malignant lung-derived cells [18,19,20]. Parallel assessment of A549 and BEAS-2B cells is therefore useful for determining whether responses observed in a cancer model are accompanied by similar or divergent effects in a non-malignant epithelial context.

Previous research has demonstrated quercetin-associated modulation of pro- and anti-apoptotic signaling in several cancer models, including changes involving *BAX*, *CASP3*, *BCL2*, and related pathways [21,22,23]. Quercetin has also been reported to modify responses to conventional chemotherapeutic agents [24,25]. From a drug-development perspective, however, a reduction in a cancer-cell readout alone is insufficient to establish selectivity; the response of non-malignant cells under comparable exposure conditions is also important when considering the biochemical profile and potential therapeutic window of a natural compound.

Comparative studies integrating concentration- and time-dependent MTT responses with targeted apoptosis-related transcriptional profiling in both A549 and BEAS-2B cells remain limited. The MTT assay reports cellular tetrazolium-reducing metabolic activity and is commonly used as a metabolism-associated cellular readout; it does not directly measure cell number, cell death, or metabolomics. qRT-PCR provides targeted information on transcriptional responses. Used together, these endpoints characterize complementary levels of cellular response to quercetin without establishing a complete mechanism of action. This approach does not constitute metabolomics, direct measurement of quercetin biotransformation, or pharmacokinetic assessment.

Therefore, the present study comparatively evaluated MTT-derived cellular metabolic activity and apoptosis-related gene expression in A549 and BEAS-2B cells across matched exposure conditions. The distinguishing feature of the design is the side-by-side assessment of malignant and immortalized non-malignant lung-derived cells over three exposure times, followed by targeted transcriptional profiling at a shared nominal quercetin concentration. This paired design permits evaluation of whether an apparent cancer-cell response is accompanied by a similar or divergent response in a non-malignant epithelial context. The objective was to characterize cell-context- and exposure-time-dependent response patterns while avoiding inference of functional apoptosis, metabolic reprogramming, or therapeutic selectivity from these endpoints alone.

## 2. Materials and Methods

### 2.1. Reagents and Cell Culture Materials

Quercetin (≥95%, HPLC; Sigma-Aldrich, St. Louis, MO, USA; Q4951), MTT reagent (M5655, Sigma-Aldrich), dimethyl sulfoxide (DMSO; D2650, Sigma-Aldrich), penicillin–streptomycin solution (100×; P4333, Sigma-Aldrich), and trypsin-EDTA (0.25%; T4049, Sigma-Aldrich) were used in all experiments. Dulbecco’s modified Eagle medium (DMEM High Glucose (DMEM-H; Capricorn Scientific, Ebsdorfergrund, Germany)), heat-inactivated fetal bovine serum (FBS; FBS-HI-11B, Capricorn Scientific), and 96-well cell culture plates (655180, Greiner Bio-One, Frickenhausen, Germany) were used for routine cell culture. All chemicals were of analytical or cell culture grade. The test article was chemically defined quercetin rather than a crude plant extract; its stated purity and product identifier are provided to support reproducibility.

### 2.2. Cell Culture

A549 (ATCC CCL-185) and BEAS-2B (ATCC CRL-9609) cell lines were obtained from the American Type Culture Collection (ATCC, Manassas, VA, USA). A549 cells are derived from human lung adenocarcinoma, whereas BEAS-2B cells are an immortalized, non-malignant human bronchial epithelial cell line originally derived from normal bronchial epithelium. Accordingly, BEAS-2B cells should be regarded as an immortalized non-malignant bronchial epithelial model rather than as primary normal bronchial epithelial cells [26]. A549 cells were cultured in Dulbecco’s modified Eagle medium (DMEM) supplemented with 10% fetal bovine serum (FBS) and 1% penicillin–streptomycin. Cells were maintained in a humidified incubator at 37 °C with 5% CO2. When cultures reached approximately 80–90% confluence, cells were passaged and cryopreserved in freezing medium containing 10% DMSO. BEAS-2B cells were cultured in DMEM supplemented with 10% FBS and 1% penicillin–streptomycin under the same incubation conditions.

### 2.3. Preparation of Quercetin Solutions

Quercetin was initially dissolved in DMSO and subsequently diluted in complete culture medium. The final DMSO concentration did not exceed 0.25% (*v*/*v*) under any experimental condition. Untreated controls and DMSO-only vehicle controls were included in parallel in the experimental design. For the MTT concentration–response experiments, the 0 µM quercetin DMSO vehicle-control condition served as the normalization reference.

### 2.4. MTT-Derived Cellular Metabolic Activity Assay

**Figure 1:**
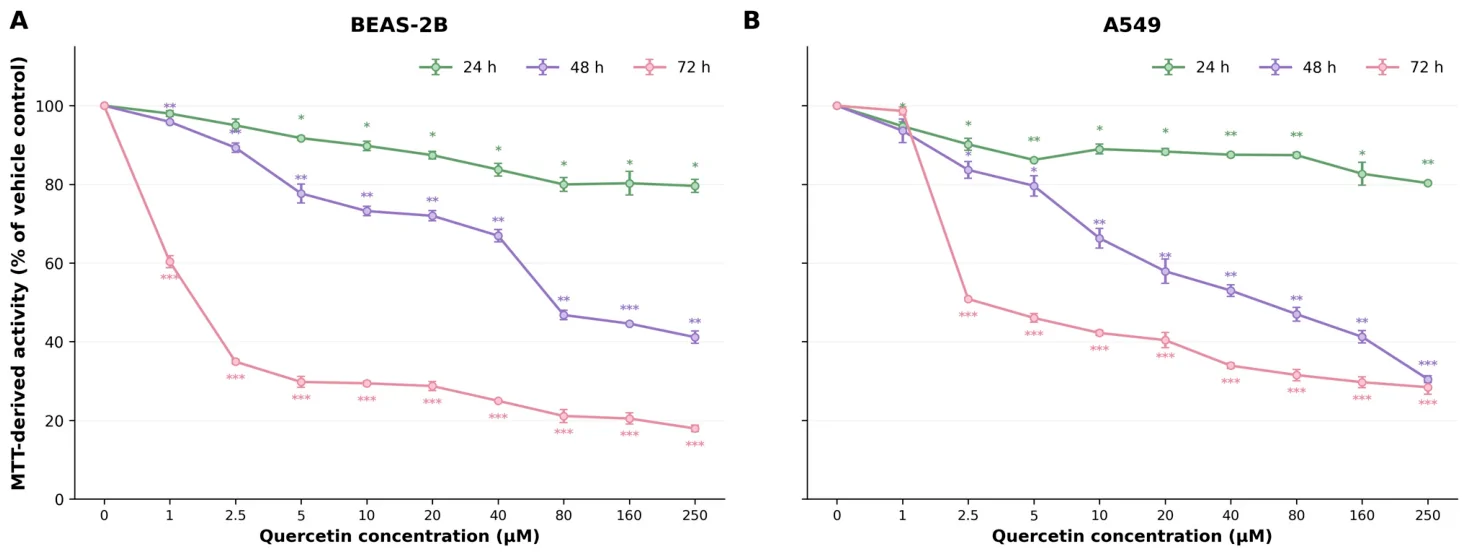
MTT-derived cellular metabolic activity in (**A**) BEAS-2B and (**B**) A549 cells after 24, 48, and 72 h of quercetin exposure (1–250 µM). Points and error bars show mean ± SD from three independent biological experiments (*n* = 3), with each experiment normalized to its own 0 µM DMSO vehicle control. Colored symbols indicate two-sided dose-versus-vehicle tests with Holm adjustment across nine doses within each cell line and exposure time (* *p* < 0.05, ** *p* < 0.01, *** *p* < 0.001). Exact unadjusted and Holm-adjusted *p*-values are provided in Supplementary Table S2A.

MTT-derived cellular metabolic activity was assessed in A549 and BEAS-2B cells. Cells were seeded into 96-well plates at 5 × 103 cells/well and exposed to quercetin at 1, 2.5, 5, 10, 20, 40, 80, 160, and 250 µM for 24, 48, and 72 h. Following exposure, the medium was removed, and 100 µL of MTT solution (0.5 mg/mL) was added to each well. Plates were incubated at 37 °C for 4 h, after which the medium was removed, and 150 µL of DMSO was used to dissolve the formazan crystals. Absorbance was measured at 570 nm using a BioTek Synergy H1 microplate reader (BioTek Instruments, Winooski, VT, USA) with Gen5 software. Three independent biological experiments were performed on separate occasions (n = 3). Within-run technical measurements were reduced to one experiment-level absorbance value per condition and were not treated as independent experimental units. For each exposure time, each biological experiment was normalized to its corresponding 0 µM quercetin DMSO vehicle-control value. The three normalized biological-replicate values were used to calculate the mean and SD shown in Figure 1 and formed the common data basis for dose-versus-vehicle testing, interaction analyses, and four-parameter logistic fitting. Supplementary File S1 provides the experiment-level absorbance values, vehicle-normalized responses, mean ± SD values, and 4PL fit summary. For each exposure period, quercetin-containing medium was applied once at the start of incubation and was not replaced or replenished before the corresponding endpoint. Mean normalized concentration–response curves calculated from the same three biological experiments were fitted using an unweighted four-parameter logistic model with free upper and lower asymptotes. The apparent MTT-derived IC50 was operationally defined as the fitted concentration at which the normalized MTT signal equaled 50% of the DMSO vehicle-control signal; this definition is distinct from the mathematical midpoint parameter of a free-asymptote four-parameter model. Model-based 95% confidence intervals for this 50%-response concentration were estimated by residual bootstrap resampling (2500 resamples). Hill slope, upper and lower asymptotes, R2, and Sy.x were retained as fit diagnostics. When the fitted response did not cross 50% within the tested concentration range, the apparent MTT-derived IC50 was reported as not determined (n.d.). This parameter is an endpoint-specific estimate of the MTT response and should not be interpreted as direct evidence of cell-death potency. A separate cell-free quercetin-plus-MTT interference control was not performed in the original experiment.

### 2.5. RNA Isolation and DNase Treatment

Total RNA was isolated using the RNeasy Mini Kit (Qiagen, Hilden, Germany; 74104) according to the manufacturer’s protocol. Genomic DNA contamination was removed using the RNase-Free DNase Set (Qiagen, 79254) during on-column purification. RNA quality and concentration were assessed with a NanoDrop spectrophotometer (Thermo Fisher Scientific, Waltham, MA, USA), and samples with A260/A280 ratios between 1.9 and 2.1 were used for downstream applications.

### 2.6. cDNA Synthesis

cDNA was synthesized from 1 µg of total RNA using the QuantiTect Reverse Transcription Kit (Qiagen, 205311), which includes a genomic DNA wipeout step. The reaction was performed according to the manufacturer’s instructions and stored at −20 °C until use.

### 2.7. Quantitative Real-Time PCR (qRT-PCR)

Gene expression analysis was performed using the QuantiTect SYBR Green PCR Kit (Qiagen, 204143) on a QuantStudio™ 3 Real-Time PCR System (Applied Biosystems, Thermo Fisher Scientific, Waltham, MA, USA). Commercially obtained primer sets routinely used in our laboratory under established qPCR conditions were used to amplify selected apoptosis-related and survival-associated genes, including *TP53*, *APAF1*, *BAX*, *BAD*, *BAK1*, *CASP2*, *CASP3*, *CASP9*, *CASP12*, *BCL2*, and *BCL2L1* (BCL-XL isoform; hereafter BCL-XL) (Table 1). *BCL2* and *BCL2L1* were treated as distinct genes. *GAPDH* served as the sole reference gene. Amplification conditions were 95 °C for 15 min, followed by 40 cycles of 94 °C for 15 s, 55–60 °C for 30 s (primer-dependent), and 72 °C for 30 s. Melting-curve analysis was used to assess product specificity.

### 2.8. Statistical Analysis

MTT experiments comprised three independent biological experiments performed on separate occasions (*n* = 3). For each exposure time and concentration, one experiment-level value from each biological experiment was normalized to the corresponding 0 µM quercetin DMSO vehicle control. These three normalized values were used to calculate the mean and SD shown in Figure 1 and formed the basis of all MTT inferential analyses. Within-run technical measurements were reduced to experiment-level values during the original data reduction and were not treated as independent experimental units. Within each cell line and exposure time, each quercetin concentration was compared with the 0 µM DMSO vehicle control within the same biological experiment using a two-sided paired t-test on the three vehicle-normalized experiment-level responses. The nine dose-versus-control *p*-values within each cell line and exposure time were adjusted using the Holm procedure. Figure 1 displays the adjusted significance thresholds, and the exact unadjusted and adjusted *p*-values are provided in Supplementary Table S2A. To evaluate whether the MTT concentration–response profile differed between cell lines, a mixed-design factorial ANOVA was performed on the experiment-level vehicle-normalized responses, with cell line as the between-group factor and quercetin concentration (1–250 µM) as the repeated factor within each biological experiment. Separate cell line × concentration interactions were tested at 24, 48, and 72 h. An overall model additionally included exposure time as a repeated factor and tested the cell line × concentration × time interaction. Because concentration and time are multi-level repeated factors and only three biological experiments were available per cell line, Greenhouse–Geisser correction was applied to the repeated-measures interaction tests rather than relying on an uncorrected sphericity assumption. Greenhouse–Geisser ε, adjusted degrees of freedom, and adjusted *p*-values are reported in Supplementary Table S2B; the interaction profiles are visualized in Supplementary Figure S1. For qRT-PCR, relative expression was determined using the 2−ΔΔCt method from three independent biological replicates (*n* = 3) per cell line and condition. The reported fold-change values, SDs, and within-cell-line inferential comparisons were calculated from these three biological replicates. Untreated, vehicle, and quercetin conditions were run in parallel; one-way ANOVA followed by Dunnett’s test was used within each cell line to evaluate quercetin-associated changes relative to the corresponding untreated control while accounting for multiple comparisons. The qRT-PCR analysis was designed for within-cell-line treatment comparisons, and no formal cell line × treatment interaction analysis was performed. Accordingly, between-cell-line transcript patterns are presented descriptively rather than as statistically established cell-line-specific treatment effects. The concentration–response reanalysis was performed in Python 3.13.5 using SciPy 1.17.0. Unweighted four-parameter logistic models were fitted to the mean normalized MTT response curves calculated from the same three biological experiments for each cell line and exposure time; model-based 95% confidence intervals for the fitted 50%-response concentration were obtained by residual bootstrap resampling (2500 resamples). R^2^ and Sy.x were used as goodness-of-fit diagnostics. The fitted-curve uncertainty reflects model-fit uncertainty of the *n* = 3 mean response curves and is not interpreted as a direct statistical comparison of fitted IC_50_ parameters between cell lines. All inferential tests were two-sided, and *p* < 0.05 was considered statistically significant. Because each biological experiment was normalized to its own 0 µM vehicle value, the vehicle reference was fixed at 100% with no residual variance; the dose-versus-vehicle paired test is therefore equivalent to a one-sample t-test of the three normalized responses against 100%. The two endpoints also differ in their reference condition: MTT responses were expressed relative to the DMSO vehicle control because vehicle normalization removes plate- and occasion-level absorbance differences between independent experiments, whereas qRT-PCR values were referenced to the untreated control in accordance with the original experimental design. DMSO-only controls were included in parallel for both endpoints, and this difference in reference condition should be considered when the two endpoints are compared.

## 3. Results

Quercetin exposure produced distinct time- and concentration-dependent changes in MTT-derived cellular metabolic activity in A549 and BEAS-2B cells.

### 3.1. Time- and Concentration-Dependent Changes in MTT-Derived Cellular Metabolic Activity

**Figure 2:**
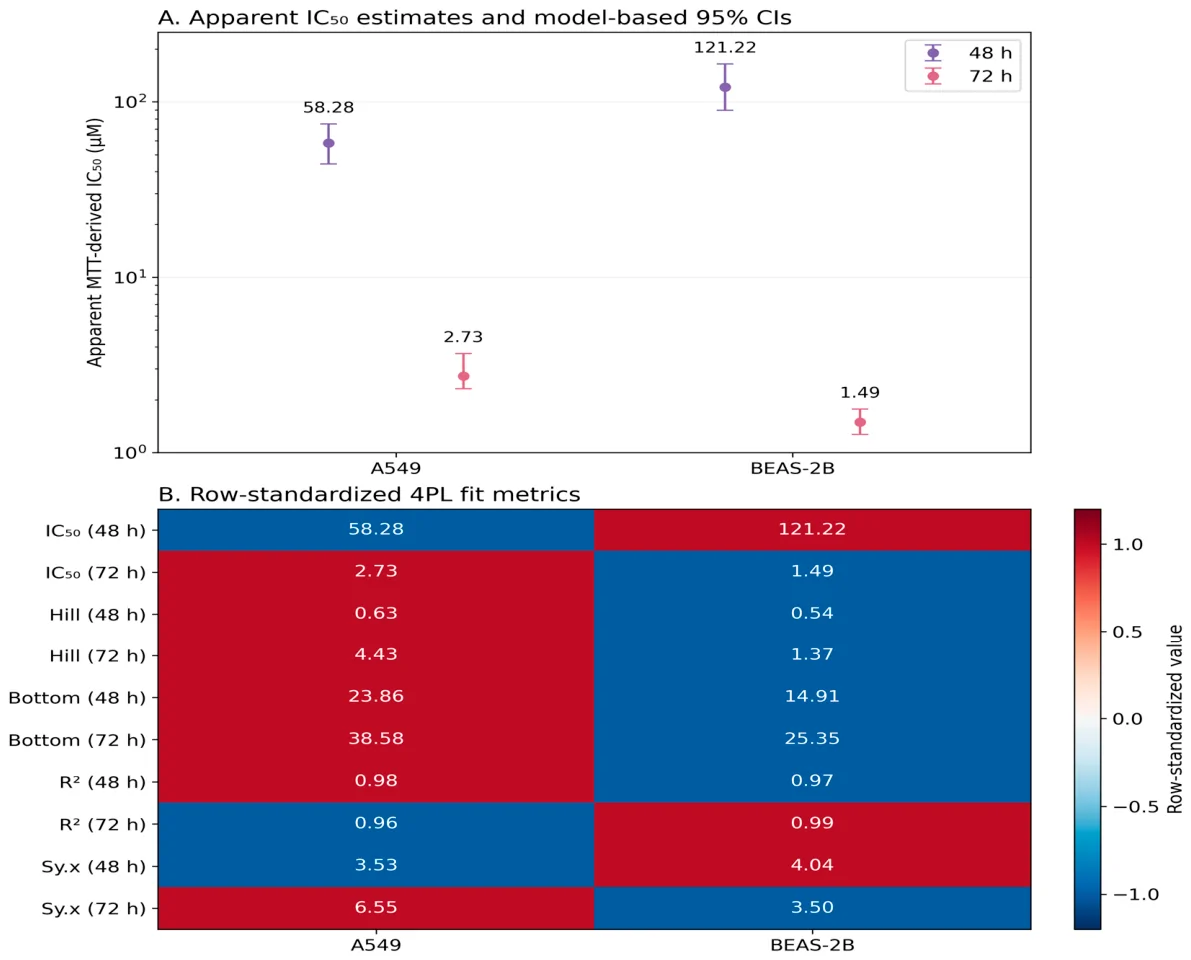
Graphical summary of the four-parameter logistic reanalysis of the MTT response using mean normalized concentration–response curves calculated from the same three independent biological experiments (*n* = 3). Panel (**A**) shows the apparent MTT-derived IC_50_ estimates at 48 and 72 h with model-based 95% confidence intervals for A549 and BEAS-2B cells. Panel (**B**) shows a heatmap of selected 4PL-derived fit metrics (row-standardized), including apparent MTT-derived IC_50_, Hill slope, lower asymptote (Bottom), R^2^, and Sy.x. At 24 h, the fitted response in both cell lines remained above 50% of vehicle control, and the apparent MTT-derived IC_50_ was therefore not determined within the tested concentration range.

Quercetin exposure resulted in a time- and concentration-dependent reduction in MTT-derived cellular metabolic activity in A549 lung adenocarcinoma cells. At 24 h, the MTT response remained above 80% across the tested concentrations, and an apparent MTT-derived IC_50_ could not be determined within the tested range. At 48 h, the fitted apparent MTT-derived IC_50_ was 58.28 µM (model-based 95% CI 44.18–74.81), with a Hill slope of 0.629, fitted upper and lower asymptotes of 101.36% and 23.86%, respectively, R^2^ = 0.983, and Sy.x = 3.53. At 72 h, the fitted apparent MTT-derived IC_50_ was 2.73 µM (2.31–3.67), with a Hill slope of 4.430, fitted upper and lower asymptotes of 101.92% and 38.58%, respectively, R^2^ = 0.958, and Sy.x = 6.55. These results show a stronger reduction in tetrazolium-reducing metabolic activity with increasing exposure duration; they do not directly quantify cell number or cell death (Figure 1 and Figure 2).

In BEAS-2B cells, quercetin also produced an exposure-time-dependent decrease in MTT-derived cellular metabolic activity. At 24 h, the MTT response remained approximately 80% or higher across the tested concentration range, and no apparent MTT-derived IC_50_ could be determined within the tested range. At 48 h, the fitted apparent MTT-derived IC_50_ was 121.22 µM (model-based 95% CI 89.50–164.66), with a Hill slope of 0.537, fitted upper and lower asymptotes of 100.49% and 14.91%, respectively, R^2^ = 0.973, and Sy.x = 4.04. At 72 h, the fitted apparent MTT-derived IC_50_ was 1.49 µM (1.27–1.77), with a Hill slope of 1.372, fitted upper and lower asymptotes of 100.16% and 25.35%, respectively, R^2^ = 0.986, and Sy.x = 3.50. Thus, prolonged exposure was associated with a substantially greater reduction in the MTT endpoint. The fitted 72 h estimate was numerically lower in BEAS-2B than in A549 cells; however, the model-based intervals describe uncertainty of the fitted mean curves rather than a formal biological between-cell-line comparison (Figure 1 and Figure 2).

### 3.2. Direct Cell Line × Concentration and Time Interaction Analysis

A mixed-design analysis of the n = 3 biological-replicate MTT responses demonstrated significant cell line × concentration interactions after Greenhouse–Geisser correction at each exposure time: 24 h, F(2.55, 10.19) = 12.61, p = 0.00119, ε = 0.318; 48 h, F(2.61, 10.45) = 30.63, p = 2.34 × 10−5, ε = 0.327; and 72 h, F(2.77, 11.08) = 109.80, p = 1.98 × 10−8, ε = 0.346. When the three exposure durations were evaluated together, the Greenhouse–Geisser-corrected cell line × concentration × time interaction was also significant, F(2.95, 11.81) = 55.95, p = 3.07 × 10−7, ε = 0.185. These findings indicate that the concentration–response profile of the MTT endpoint differed between A549 and BEAS-2B cells and that the magnitude and direction of this difference changed with exposure duration. The interaction analysis tests the experiment-level MTT response profiles; it does not constitute a direct statistical comparison of the fitted apparent MTT-derived IC50 parameters and does not by itself establish differential cell-death sensitivity or a therapeutic window. Given the small number of biological experiments, these interaction results should be interpreted cautiously. The interaction profiles are visualized in Supplementary Figure S1, and the corrected interaction statistics are provided in Supplementary Table S2B.

### 3.3. Gene Expression Changes in BEAS-2B and A549 Cells

**Figure 3:**
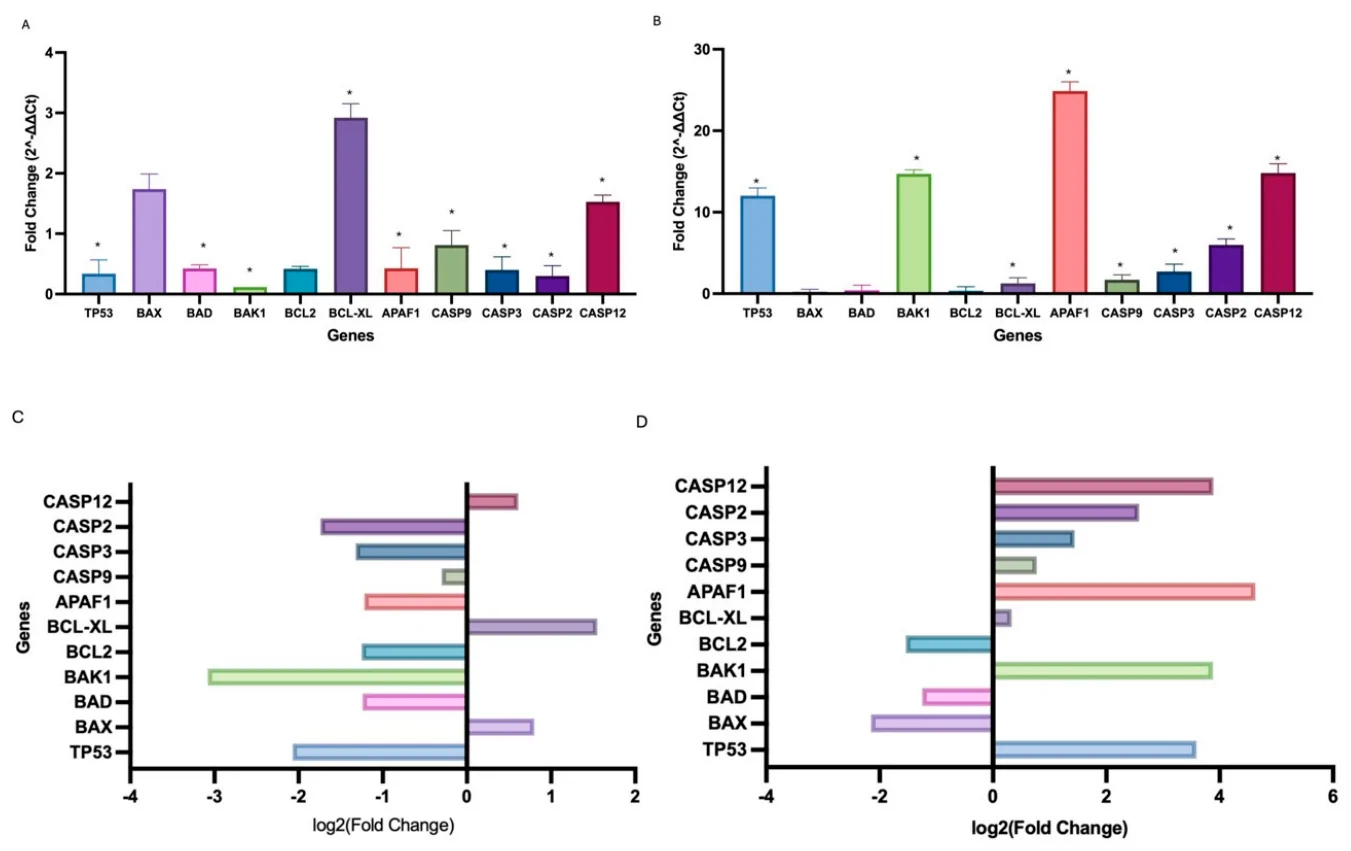
Apoptosis-related transcriptional responses to quercetin (45 µM, 48 h). Relative expression (2^−ΔΔCt^) is shown for (**A**) BEAS-2B and (**B**) A549 cells; (**C**,**D**) show the corresponding log_2_(fold change) values. Data in panels A and B are the mean ± SD from three independent biological replicates (*n* = 3). DMSO-only vehicle controls were included in parallel but are not displayed. Within each cell line, the reported comparison with untreated control used one-way ANOVA followed by Dunnett’s test (* *p* < 0.05). BCL-XL denotes the *BCL2L1*-encoded BCL-XL isoform.

For transcriptional analyses, both cell lines were exposed to 45 µM quercetin for 48 h to permit comparison under the same nominal exposure. The 45 µM concentration was not included as a discrete concentration in the MTT series. In the four-parameter reanalysis, 45 µM was below the fitted 48 h apparent MTT-derived IC_50_ estimates for both A549 (58.28 µM) and BEAS-2B (121.22 µM) cells and was close to the directly tested 40 µM condition. The matched nominal concentration was therefore retained as the exposure used in the original qRT-PCR experiment, but it should not be interpreted as a biologically equivalent or equitoxic exposure in the two cell lines. This approach is consistent with previous comparative work using common nominal quercetin concentrations in BEAS-2B, A549, and H1299 cells [19]. Accordingly, the qRT-PCR results are interpreted as apoptosis-related transcriptional responses under a shared exposure condition rather than as evidence of functional apoptosis. In BEAS-2B cells, *BCL2L1* (BCL-XL) showed the largest increase (2.922-fold), followed by *BAX* (1.739-fold) and *CASP12* (1.528-fold). *BAK1*, *CASP2*, *TP53*, *CASP3*, *BCL2*, *BAD*, and *APAF1* were reduced, and *CASP9* also showed a decrease. These observations describe the direction and magnitude of transcript changes relative to the corresponding untreated control (Figure 3A). Relative to the untreated control, the changes in *TP53*, *BAD*, *BAK1*, *BCL2L1* (BCL-XL), *APAF1*, *CASP9*, *CASP3*, *CASP2*, and *CASP12* were significant in the within-cell-line one-way ANOVA with Dunnett’s test (*p* < 0.05), whereas the changes in *BAX* and *BCL2* were not (Figure 3A).

In A549 cells, quercetin exposure was associated with large transcript-level changes in several of the evaluated genes. *APAF1* showed the highest increase (24.888-fold), followed by *CASP12* (14.811-fold), *BAK1* (14.718-fold), *TP53* (12.026-fold), *CASP2* (5.996-fold), *CASP3* (2.716-fold), and *CASP9* (1.701-fold). *BCL2L1* (BCL-XL) increased 1.256-fold, whereas *BAX* (0.226-fold), *BAD* (0.422-fold), and *BCL2* (0.344-fold) were reduced. These results indicate broad modulation of apoptosis-related transcripts but do not establish functional activation or execution of apoptosis. The simultaneous downregulation of *BAX* and *BAD* also shows that the pattern is not uniformly consistent with a canonical mitochondrial apoptotic program. *CASP12* upregulation is retained only as an exploratory transcript-level observation and is not interpreted as evidence of ER-stress-mediated apoptosis because CASP4 and canonical upstream endoplasmic-reticulum-stress markers were not assessed (Figure 3B). In the within-cell-line one-way ANOVA with Dunnett’s test, the changes in *TP53*, *BAK1*, *BCL2L1* (BCL-XL), *APAF1*, *CASP9*, *CASP3*, *CASP2*, and *CASP12* were significant relative to the untreated control (*p* < 0.05), whereas the changes in *BAX*, *BAD*, and *BCL2* were not (Figure 3B).

Descriptive comparison of the two expression profiles showed different response patterns under the matched 45 µM, 48 h exposure. In A549 cells, *APAF1*, *BAK1*, *TP53*, *CASP2*, *CASP3*, and *CASP9* were increased relative to the untreated control, whereas *BAX*, *BAD*, and *BCL2* were reduced. In BEAS-2B cells, *BCL2L1* (BCL-XL) and *BAX* increased, whereas *BAK1*, *CASP2*, *TP53*, *CASP3*, *BCL2*, *BAD*, and *APAF1* decreased. *CASP12* increased in both cell lines but is interpreted cautiously. The qRT-PCR analysis was designed for within-cell-line treated-versus-control comparisons using three independent biological replicates (*n* = 3), and no formal cell line × treatment interaction analysis was performed. Therefore, cross-cell-line differences are presented as descriptive patterns rather than statistically established cell-line-specific treatment effects (Figure 3C,D; Table 2).

### 3.4. Integrated Comparison of Apoptosis-Related Transcriptional Responses

**Figure 4:**
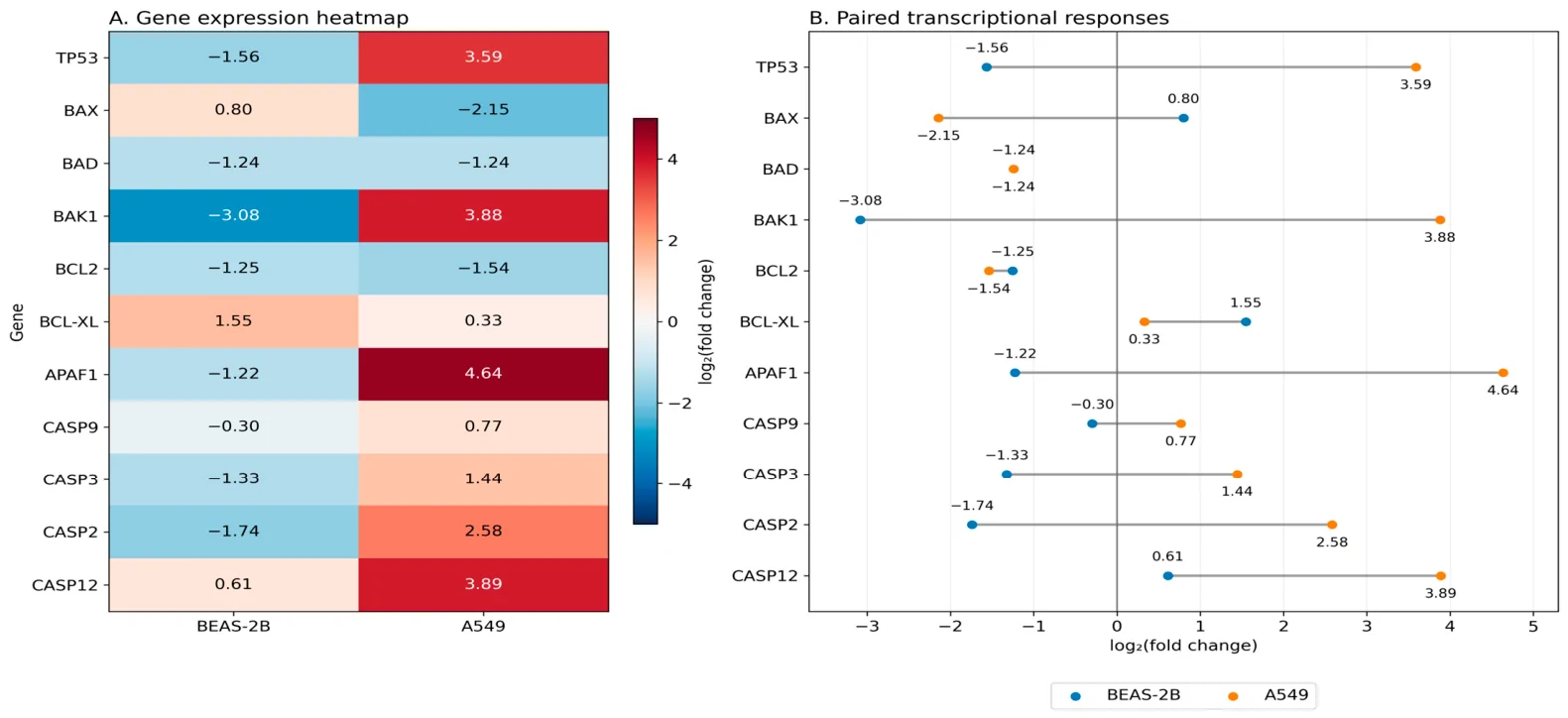
Descriptive integrated comparison of apoptosis-related transcriptional responses to quercetin (45 µM, 48 h). (**A**) Gene-wise log_2_(fold change) heatmap and (**B**) paired plot for BEAS-2B and A549 cells. In panel A, blue and red shading indicate negative and positive log2(fold change) values, respectively; color intensity represents magnitude. In panel B, blue points represent BEAS-2B and orange points represent A549; gray lines connect the two cell-line values for each gene. Positive and negative values denote up- and downregulation, respectively. Values are derived from Table 2; the figure is descriptive and is not an independent statistical test. BCL-XL in the figure denotes the *BCL2L1* transcript encoding the BCL-XL isoform.

To facilitate visual comparison between the two cell lines, log_2_-transformed fold-change values were summarized as a gene-wise heatmap and paired plot (Figure 4). The descriptive visualization shows opposite directions for *TP53*, *BAK1*, *APAF1*, *CASP2*, *CASP3*, and *BAX* across the two cell lines, whereas *BAD* and *BCL2* decreased in both and *BCL2L1* (BCL-XL) increased in both. *CASP12* also increased in both cell lines but is interpreted as exploratory. Figure 4 is an integrative display of the same qRT-PCR dataset, not an independent statistical test or mechanistic assay. Because the qRT-PCR analysis was designed for within-cell-line treatment comparisons and no formal cell line × treatment interaction analysis was performed, no inferential claim of a cell-line-specific treatment effect is made from this comparison.

## 4. Discussion

Quercetin is a secondary plant metabolite with broad biochemical activity and sustained interest in anticancer research. In this study, quercetin produced concentration- and exposure-time-dependent changes in the MTT response in both A549 and BEAS-2B cells. Four-parameter logistic reanalysis of the mean normalized concentration–response curves calculated from three independent biological experiments yielded apparent MTT-derived IC_50_ estimates of 58.28 µM (95% CI 44.18–74.81) for A549 and 121.22 µM (89.50–164.66) for BEAS-2B cells at 48 h. At 72 h, the corresponding estimates were 2.73 µM (2.31–3.67) and 1.49 µM (1.27–1.77). Thus, the numerical ordering of the fitted point estimates differed between 48 and 72 h. The model-based confidence intervals quantify uncertainty of the fitted mean curves and are not a direct statistical test of IC_50_ differences between cell lines. The experiment-level mixed-design analysis, with Greenhouse–Geisser correction for the multi-level repeated factors, demonstrated significant cell line × concentration interactions at 24, 48, and 72 h and a significant cell line × concentration × time interaction, supporting a difference in the MTT response profiles between cellular contexts and across exposure duration. Because only three independent biological experiments were available per cell line, these interaction findings should be interpreted cautiously and do not establish a stable cancer-selective effect or a therapeutic window. Quercetin-containing medium was not replenished during the 72 h exposure, and quercetin is known to degrade in culture medium and generate hydrogen peroxide over time [27,28]. Degradation products and/or secondary reactive species may therefore have contributed to the long-exposure response, but this remains a hypothesis because these species were not measured. In addition, MTT reduction reflects cellular tetrazolium-reducing metabolic activity rather than cell number or cell death directly, and quercetin can reduce MTT in the absence of viable cells [29,30,31,32]. The long-exposure findings should therefore be interpreted as changes in an MTT-based metabolic response rather than definitive cytotoxicity.

For transcriptional analysis, the same nominal quercetin concentration (45 µM) was applied to both cell lines for 48 h. This concentration was not tested as a discrete point in the MTT series. In the four-parameter logistic analysis, 45 µM was below the fitted apparent MTT-derived IC_50_ point estimates for both cell lines at 48 h and remained close to the directly tested 40 µM condition; numerically, it was closer to the A549 estimate than to the BEAS-2B estimate. The design was intended to compare the two cellular contexts under a matched nominal exposure, not to create biologically equivalent or equitoxic conditions. This approach is consistent with comparative work using common quercetin concentrations in BEAS-2B, A549, and H1299 cells [19]. The qRT-PCR profiles should therefore be interpreted as transcriptional responses to a shared exposure rather than as proof of apoptosis or a direct measure of cellular metabolism.

The inclusion of a non-malignant comparator is central to the interpretation of these data. A reduction in an MTT endpoint in a cancer cell line alone cannot establish a favorable therapeutic window. In the four-parameter logistic analysis, the A549 fitted apparent MTT-derived IC_50_ point estimate was lower than the BEAS-2B estimate at 48 h, whereas at 72 h BEAS-2B had the lower point estimate. The direct interaction analysis complements this descriptive IC_50_ pattern by showing that the overall MTT concentration-response profiles differed significantly between the two cell lines at each exposure time and that this difference itself changed across time. Nevertheless, interaction of the MTT profiles should not be equated with selective cytotoxicity: the fitted IC_50_ values remain endpoint-specific estimates, the direction of the cell-line difference is not stable across time, and the assay does not directly quantify cell death. Accordingly, a stable cancer-selective response or therapeutic window cannot be inferred from the tested conditions.

The interaction pattern is consistent with published evidence that quercetin responses are strongly dependent on both exposure duration and cellular context. Hashemzaei et al. reported progressively greater MTT inhibition with longer quercetin exposure across a diverse cell-line panel and substantial variation in apparent sensitivity among cell models [12]. In lung-cancer models, Mukherjee and Khuda-Bukhsh observed different quercetin responses in A549 and H460 cells, with A549 showing a stronger MTT response at lower concentrations, further illustrating cell-line dependence [20]. Comparative work by Zhou et al. used common nominal quercetin exposures across malignant and non-malignant lung-derived models and linked the response to SIRT5/PI3K/AKT and DNA-damage signaling, supporting the concept that the same nominal exposure can produce context-dependent effects [19]. In BEAS-2B cells, quercetin has also been shown to activate ERK1/2-dependent Nrf2/HO-1 signaling under oxidative-stress conditions, indicating that non-malignant bronchial epithelial cells can engage adaptive redox-response programs [33]. Zhaorigetu et al. likewise reported exposure-time-dependent quercetin effects in A549 cells together with a normal-cell comparator [34]. These reports support interpretation of the present interaction as a cell-context- and time-dependent difference in the MTT response, while not establishing universal cancer selectivity.

From a metabolism-focused perspective, the MTT endpoint captures cellular tetrazolium-reducing activity, which is influenced by redox and metabolic state. It does not identify specific metabolic pathways, quantify intracellular metabolites, or determine quercetin biotransformation. The metabolic relevance of the present study therefore lies in characterizing a metabolism-associated cellular phenotype produced by a defined secondary plant metabolite, not in claiming metabolomics or metabolic reprogramming. Direct pathway-level conclusions would require targeted or untargeted metabolomics together with measurement of quercetin and its transformation products. This distinction defines both the scope of the findings and their relevance to natural-product and drug-metabolism research.

In BEAS-2B cells, the dominant transcriptional increase involved *BCL2L1* (BCL-XL), which encodes an anti-apoptotic/pro-survival isoform [35], together with downregulation of *BAK1*, *CASP2*, *TP53*, *CASP3*, *BCL2*, *BAD*, and *APAF1*. *BCL2* and *BCL2L1* are distinct genes and are treated separately throughout the revised manuscript. The overall BEAS-2B profile does not resemble uniform activation of a classical apoptotic cascade and may reflect a stress-adaptive response. Previous work has shown that quercetin can activate ERK1/2-dependent Nrf2/HO-1 signaling in BEAS-2B cells under oxidative stress [33]. Because BCL-XL protein abundance and survival-associated signaling were not directly measured here, a protective interpretation remains inferential and requires functional validation.

In A549 cells, quercetin exposure was associated with increased *APAF1*, *BAK1*, *TP53*, *CASP2*, *CASP3*, and *CASP9* transcripts, alongside reduced *BCL2*, *BAX*, and *BAD*. Although several of the increased transcripts participate in apoptosis-related pathways, the concurrent downregulation of *BAX* and *BAD* means that the response is not uniformly aligned with a canonical mitochondrial pro-apoptotic program. *BAX* function depends on conformational activation and mitochondrial translocation, whereas *BAD* activity is strongly regulated by phosphorylation [36,37]. The present data therefore support cell-context-dependent modulation of apoptosis-related transcription rather than a transcript-based conclusion that apoptosis was induced. Such variability in quercetin responses across cancer models has been reported more broadly [34,38,39,40,41].

*CASP12* transcript levels increased in both cell lines, more markedly in A549 cells, but this observation should be interpreted cautiously. Unlike the fully functional murine gene, human *CASP12* commonly carries a nonsense polymorphism that renders the protein largely non-functional in most individuals [42]. CASP4 and canonical unfolded-protein-response markers were not assessed [43,44], so an endoplasmic-reticulum-stress mechanism cannot be inferred. More broadly, quercetin has been reported to influence p53, NF-κB, MAPK, PI3K/AKT, oxidative-stress, mitochondrial, and DNA-damage-related processes [45,46,47,48]. Recent work combining transcript-level analysis with Annexin V/PI staining, ROS measurement, mitochondrial membrane-potential assessment, and three-dimensional models illustrates the additional evidence needed to progress from transcriptional association to mechanistic interpretation [49]. Functional confirmation in the present models would therefore require assays such as Annexin V/PI flow cytometry, caspase activity measurements, TUNEL analysis, or detection of cleaved caspases and PARP [20,50].

The integrated comparison in Figure 4 further illustrates that the transcriptional response was neither uniform across cell lines nor uniformly aligned with a single apoptotic direction. Several genes changed in opposite directions, whereas *BAD* and *BCL2* decreased in both cell lines, and *BCL2L1* (BCL-XL) increased in both.

Taken together, the paired A549-BEAS-2B design shows that quercetin produces exposure-time-dependent changes in the MTT endpoint and distinct apoptosis-related transcriptional profiles in malignant and immortalized non-malignant lung-derived cells. The side-by-side design is informative because it shows that an apparent cancer-cell response is not accompanied by a stable cancer-selective pattern across time. Future work integrating orthogonal cell-response assays, functional apoptosis measurements, protein-level validation, targeted or untargeted metabolomics, and direct analysis of quercetin stability and biotransformation would provide a more complete framework linking exposure, metabolism-associated cellular responses, molecular signaling, and antineoplastic potential.

### Study Limitations

Several limitations should be considered when interpreting these findings. Cell response was assessed only with the MTT assay, which reflects tetrazolium-reducing metabolic activity rather than cell number, proliferation, or death directly. Quercetin can also reduce MTT independently of viable cells [29,30,31,32], and a separate cell-free quercetin-plus-MTT interference control was not included in the original experiment; direct chemical interference therefore cannot be excluded. For the MTT experiments, the 0 µM quercetin DMSO vehicle-control values from all three independent biological experiments are provided in Supplementary File S1 and were used as the normalization reference. A separate untreated-versus-vehicle significance test is not reported, and the study therefore does not claim that DMSO itself had no effect relative to untreated cultures. For qRT-PCR, DMSO-only vehicle controls were included in parallel but are not displayed; no separate vehicle-versus-untreated inferential claim is made. The exposure medium was not replenished during the 72 h incubation, and the marked decrease in apparent MTT-derived IC_50_ estimates at 72 h should be interpreted cautiously in the absence of an orthogonal assay such as trypan blue exclusion, LDH release, ATP-based viability, clonogenic survival, or live/dead imaging. The four-parameter confidence intervals were obtained from residual bootstrap analysis of mean normalized concentration–response curves calculated directly from the same three biological experiments used for Figure 1 and inferential analyses; they describe model-fit uncertainty and do not constitute direct tests of fitted IC_50_ differences between cell lines. In addition, the fitted 72 h transition occurred within the lowest tested concentrations (1–5 µM), so the 72 h apparent MTT-derived IC_50_ estimates are less well constrained by the concentration grid than the 48 h estimates, and their model-based intervals should be interpreted accordingly. The mixed-design interaction analysis used the experiment-level MTT response profiles and Greenhouse–Geisser correction for the multi-level repeated factors. Although the corrected cell line × concentration and cell line × concentration × time interactions were significant, these tests remain limited by the small number of independent biological experiments (*n* = 3 per cell line) and by the MTT-specific limitations described above; they should not be interpreted as proof of differential cell-death sensitivity. Transcriptional analysis was performed at a single shared nominal concentration (45 µM) and a single exposure time (48 h); 45 µM was not a discrete concentration in the MTT series and does not represent an equitoxic exposure in the two cell lines. No protein-level or functional apoptosis assay was performed, and CASP12 was evaluated only as an exploratory transcript without CASP4 or canonical unfolded-protein-response markers. Gene expression was normalized to the single reference gene GAPDH; an additional reference gene was not included, and GAPDH stability under quercetin exposure was not independently validated. The primer sets were commercially obtained and routinely used under established laboratory qPCR conditions; however, primer-specific amplification efficiencies were not independently determined by standard-curve analysis within this study. The qRT-PCR analysis was based on three independent biological replicates per cell line and condition and was designed for within-cell-line treated-versus-control comparisons. Because no formal cell line × treatment interaction analysis was performed, cross-cell-line transcript differences are interpreted descriptively. The relatively small number of biological replicates (*n* = 3) may also limit sensitivity to subtle effects. Finally, the study did not perform targeted or untargeted metabolomics, quantify intracellular or extracellular quercetin, identify quercetin metabolites, or measure degradation products in the culture medium. Thus, the MTT findings represent a metabolism-associated cellular response rather than direct evidence of metabolic pathway reprogramming or drug metabolism. Protein-level, functional, metabolomic, pharmacokinetic, and in vivo validation will be required before translational conclusions can be drawn.

## 5. Conclusions

Quercetin produced exposure-time-dependent changes in MTT-derived cellular metabolic activity and apoptosis-related transcriptional responses in A549 and BEAS-2B cells. For the MTT endpoint, Greenhouse–Geisser-corrected mixed-design analysis showed significant cell line × concentration interactions at 24, 48, and 72 h and a significant cell line × concentration × time interaction, indicating that the concentration–response profiles differed between the two cell models and changed with exposure duration. Four-parameter reanalysis of the same n = 3 biological-replicate mean curves also yielded different numerical ordering of the apparent MTT-derived IC50 point estimates at 48 and 72 h, with the lower 72 h point estimate in BEAS-2B cells. Because the direction of the difference was not stable across time, the interaction analyses were based on only three biological experiments per cell line, and because MTT does not directly measure cell death, these findings do not establish a stable cancer-selective response or a clear therapeutic window. At 45 µM for 48 h, the two cell lines displayed different transcript-level patterns. The qRT-PCR results were derived from three independent biological replicates and analyzed within each cell line; because no formal cell line × treatment interaction analysis was performed, cross-cell-line transcriptional differences remain descriptive.

These findings provide a comparative biochemical characterization of quercetin by linking an MTT-based, tetrazolium-reducing metabolic response with targeted apoptosis-related transcriptional profiling in malignant and immortalized non-malignant lung-derived cells. The data do not establish cell death, functional apoptosis, metabolic pathway reprogramming, or clinical selectivity. Orthogonal cell-response and apoptosis assays, protein-level validation, metabolomic profiling, and direct analysis of quercetin stability and biotransformation are required to extend these observations mechanistically and translationally.

## Figures and Tables

**Table 1:** Primer sequences used for qRT-PCR analysis.

Gene	Forward Primer (5′–3′)	Reverse Primer (5′–3′)	Amplicon Size (bp)
*TP53*	CAGCACATGACGGAGGTTGT	TCATCCAAATACTCCACACGC	125
*APAF1*	AAGGTGGAGTACCACAGAGG	TCCATGTATGGTGACCCAT	116
*BAX*	CCCGAGAGGTCTTTTTCCGAG	CCAGCCCATGATGGTTCTGAT	155
*BAD*	CCCAGAGTTTGAGCCGAGTG	CCCATCCCTTCGTCGTCCT	249
*BAK1*	CATCAACCGACGCTATGACTC	GTCAGGCCATGCTGGTAGAC	192
*BCL2*	GGTGGGGTCATGTGTGTGG	CGGTTCAGGTACTCAGTCATCC	89
*BCL2L1*	GAGCTGGTGGTTGACTTTCTC	TCCATCTCCGATTCAGTCCCT	119
*CASP2*	AGCTGTTGTTGAGCGAATTGT	AGCAAGTTGAGGAGTTCCACA	124
*CASP3*	CATGGAAGCGAATCAATGGACT	CTGTACCAGACCGAGATGTCA	139
*CASP9*	CTCAGACCAGAGATTCGCAAAC	GCATTTCCCCTCAAACTCTCAA	116
*CASP12*	AACAACCGTAACTGCCAGAGT	CTGCACCGGCTTTTCCACT	118
*GAPDH*	GGAGCGAGATCCCTCCAAAAT	GGCTGTTGTCATACTTCTCAT	197

**Table 2:** Comparative fold-change summary of apoptosis-related genes in BEAS-2B and A549 cells after quercetin treatment (45 µM, 48 h).

Gene	BEAS-2B Fold Change	BEAS-2B Expression Pattern	A549 Fold Change	A549 Expression Pattern
*TP53*	0.338	Downregulated	12.026	Upregulated
*BAX*	1.739	Upregulated	0.226	Downregulated
*BAD*	0.423	Downregulated	0.422	Downregulated
*BAK1*	0.118	Downregulated	14.718	Upregulated
*BCL2*	0.419	Downregulated	0.344	Downregulated
*BCL2L1*	2.922	Upregulated	1.256	Upregulated
*APAF1*	0.428	Downregulated	24.888	Upregulated
*CASP9*	0.813	Downregulated	1.701	Upregulated
*CASP3*	0.399	Downregulated	2.716	Upregulated
*CASP2*	0.299	Downregulated	5.996	Upregulated
*CASP12*	1.528	Upregulated; interpreted cautiously	14.811	Upregulated; interpreted cautiously

Expression-pattern labels indicate the direction of change relative to the untreated control; the magnitude of change should be interpreted from the corresponding fold-change values.

## Data Availability

The data presented in this study are contained within the article, Supplementary File S1, Supplementary Table S2A–C, and Supplementary Figure S1. Supplementary File S1 provides the experiment-level MTT absorbance values and vehicle-normalized responses from the three independent biological experiments used to calculate Figure 1 means and SDs, dose-versus-vehicle statistics, interaction analyses, and 4PL mean curves; Supplementary Table S2A–C provides the corresponding dose-versus-vehicle statistics, Greenhouse–Geisser-corrected interaction statistics, and 4PL fit diagnostics. Additional retained analysis files are available from the corresponding author upon reasonable request.

## Supplementary Materials

The following supporting information can be downloaded at: https://www.mdpi.com/article/10.3390/metabo16100740/s1. Supplementary File S1 provides the experiment-level MTT absorbance values, vehicle-normalized responses, mean ± SD values from three independent biological experiments (n = 3), and the corresponding 4PL fit summary. Supplementary Table S2A provides the same n = 3 mean MTT responses, SD values, and exact two-sided dose-versus-vehicle p-values with Holm adjustment across nine concentrations within each cell line and exposure time. Supplementary Table S2B reports the Greenhouse–Geisser-corrected mixed-design interaction analyses (cell line × concentration at 24, 48, and 72 h and the overall cell line × concentration × time interaction), including ε and adjusted degrees of freedom. Supplementary Table S2C summarizes the 4PL fit diagnostics. Supplementary Figure S1 provides an interaction-profile plot and heatmap based on the same n = 3 biological-replicate MTT responses.
